# A foundation model-driven multi-view collaborative framework for semi-supervised 3D medical image segmentation

**DOI:** 10.3389/fmed.2025.1744097

**Published:** 2026-01-12

**Authors:** Lina Li, Bin Wang, Hong Zhang

**Affiliations:** 1 Department of Radiology, First Hospital of Shanxi Medical University, Taiyuan, China; 2 Department of Medical Imaging, Shanxi Medical University, Taiyuan, China; 3 Department of Nuclear Medicine and Medical PET Center, The Second Hospital of Zhejiang University School of Medicine, Hangzhou, China

**Keywords:** foundation model, medical image segmentation, multi-view learning, segment anything model, semi-supervised learning

## Abstract

**Background:**

3D medical image segmentation is a cornerstone for quantitative analysis and clinical decision-making in various modalities. However, acquiring high-quality voxel-level annotations is both time-consuming and labor-intensive. Semi-supervised learning (SSL) provides an appealing solution by effectively utilizing limited labeled data along with abundant unlabeled data to enhance segmentation performance under clinical data constraints.

**Methods:**

We propose a foundation model-driven multi-view collaborative learning framework that exploits zero-shot capabilities of SAM-like foundation models to jointly learn from axial, sagittal, and coronal planes. A collaborative fusion module integrates complementary representations across views, enhancing 3D structural understanding and improving the performance with limited annotation cost.

**Results:**

Extensive experiments on two evaluation datasets including MRI brain tumor segmentation and whole-body PET heart segmentation demonstrate that our proposed method consistently outperforms existing SAM-based semi-supervised approaches. The multi-view collaborative design not only refines boundary precision for organ and tumor delineation but also shows strong transferability across imaging modalities.

**Conclusion:**

This study presents a foundation model-driven, multi-view collaborative learning paradigm that efficiently advances semi-supervised 3D medical image segmentation, which provides a scalable and clinically meaningful solution that reduces annotation dependency while maintaining high segmentation accuracy across diverse medical imaging modalities.

## 1 Introduction

Three-dimensional (3D) medical imaging is widely used for creating cross-sectional volumetric images within various body regions, which plays a fundamental role in quantitative analysis, computer-assisted diagnosis, and treatment planning across diverse imaging modalities (1, 2). It enables the delineation of anatomical structures and pathological regions within volumetric data, facilitating accurate disease localization and progression monitoring (3, 4). For example, brain tumor segmentation from magnetic resonance imaging (MRI) is crucial step to determine the location and size of tumor areas, which plays a vital role in surgical planning, treatment design, efficacy evaluation, and longitudinal monitoring (5, 6). The complexity of brain tumors, characterized by significant heterogeneity in invasiveness, prognosis, and tissue characteristics, poses a challenge to accurate segmentation (7). In addition to MRI, positron emission tomography (PET) has emerged as another important 3D imaging modality that provides quantitative insights into physiological and metabolic processes (8, 9). PET heart segmentation plays a key role in cardiac function assessment, lesion detection, and treatment response evaluation, enabling clinicians to localize metabolic abnormalities and measure tissue activity in a noninvasive manner.

Existing clinical standards rely on the manual delineation of tumor or lesion boundaries by expert radiologists and radiation oncologists, which is not only exceptionally time-consuming and labor-intensive but also subject to significant inter- and intra-observer variability, potentially affecting the consistency of patient care. The clear need for automated, objective, and efficient segmentation tools has driven extensive research into deep learning-based solutions. Recently, deep learning-based segmentation methods have shown significant improvements for many medical image segmentation tasks (10–12). However, the development of robust and generalizable deep learning models is fundamentally constrained by a persistent data bottleneck. These models typically require vast amounts of high-quality, pixel-wise annotated data for training, which are often costly and time-consuming to collect (13). Manually annotating medical images at the pixel level is a costly and time-consuming process that requires the expertise of experienced clinical professionals, which significantly hinders the practical deployment of medical image segmentation models in real-world scenarios (14).

To address this challenge, semi-supervised medical image segmentation (SSMIS) has emerged as a compelling paradigm to address this data scarcity challenge (15). SSMIS aims to build accurate segmentation models by leveraging a small amount of labeled data in conjunction with a large amount of unlabeled data.

Most state-of-the-art SSMIS methods rely on two foundational schemes: pseudo labeling and unsupervised regularization. For pseudo labeling, the core is generating reliable pseudo labels for unlabeled data from pre-trained or dynamically updated models and using them as weakly supervised data (16–18) with quality-control strategies like confidence thresholding (19, 20) and adaptive refinement (21, 22). In contrast, unsupervised regularization does not generate pseudo labels but imposes invariance constraints to learn robust features from unlabeled data (23–26). One commonly used strategy is consistency learning by enforcing consistent predictions for perturbed unlabeled images (27–30). However, the performance of these methods inherently depending on the knowledge transfer from labeled to unlabeled data. As such, most methods struggles to achieve satisfactory outcomes when faced with extremely limited labeling budgets.

Concurrently, the field of artificial intelligence has been revolutionized by the advent of large-scale, pre-trained foundation models. In computer vision, the Segment Anything Model (SAM) (31) represents a landmark achievement. Trained on an unprecedented scale of over one billion masks from 11 million natural images, SAM exhibits remarkable capabilities for zero-shot and few-shot generalization across a diverse range of segmentation tasks. Recently, some approaches have attempted to integrate SAM (31) into SSMIS frameworks. Several medical SAM adaptions are also emerged to enhance the performance on medical images due to the distribution gap between natural images and medical images (32, 33). Despite SAM requires prompts for interactive segmentation, integrating SAM into SSMIS frameworks can build a robust automatic segmentation framework that effectively utilizes unlabeled data using foundation models (34, 35). Existing approaches mainly generate point prompts from prediction regions of SSMIS to enable automatic segmentation using SAM. Despite these efforts, pseudo labels often contain noisy regions, and directly generating prompts from such pseudo labels can introduce inaccuracies in the learning procedure (36).

In the realm of 3D medical imaging, the image data is inherently multi-dimensional different from 2D images, which can be utilized to capture anatomical structures from various perspectives. Leveraging this multi-view information is crucial for several reasons. Firstly, different views including axial, coronal, and sagittal provide complementary information about the volumetric relationships and morphological characteristics of anatomical structures. Fusing these views can lead to a more comprehensive understanding of the underlying anatomy, which is essential for accurate segmentation (37). Besides, multi-view fusion can mitigate the impact of noise and artifacts that may be present in one view but not in others. By integrating information from multiple perspectives, the model can become more robust to such imperfections, leading to more reliable segmentation and analysis (38).

In this paper, we propose a foundation model-driven multi-view collaborative learning framework that extends SAM-like foundation models to jointly learn from axial, sagittal, and coronal planes for fusion of different features to enhances the learning process. We aim to enhance the quality of pseudo labels to improve the overall performance in the learning procedure. Its core innovation lies in the first-time extension of SAM-like foundation models to multi-view (axial, sagittal, and coronal) collaborative learning for 3D medical images. We validate the effectiveness of our framework on two evaluation datasets including MRI brain tumor segmentation (39) and whole-body PET heart segmentation (9). The experimental results demonstrate our proposed method significantly improves the performance for 3D medical segmentation of existing SAM-based semi-supervised methods, which highlight the potential of our framework to advance treatment planning with minimal manual annotation efforts for developing artificial intelligence algorithms.

## 2 Materials and methods

### 2.1 Task definition

In the semi-supervised 3D medical image segmentation (SSMIS) task, the dataset consists of two parts: a small set of labeled images and a large set of unlabeled images. The labeled subset provides voxel-wise annotations that identify specific anatomical or pathological structures, such as brain tumors in MRI scans. These annotations are essential for guiding supervised learning but are limited in number due to the high cost and expertise required for manual labeling. In contrast, the unlabeled subset contains a large number of medical images without annotations, which still carry rich spatial and contextual information that can be exploited to improve model generalization.

The goal of the SSMIS task is to develop a segmentation model that can accurately identify target regions by jointly learning from both labeled and unlabeled data. Let *x* denote a 3D medical image and ŷ = *f*_θ_(*x*) represent the predicted segmentation map generated by a model *f*_θ_ parameterized by θ. The model is optimized by combining the supervised learning objective on labeled data with a regularization or consistency term on unlabeled data, formulated as:


$$\begin{array}{l} L_{\text{total}}=L_{\text{sup}}+\lambda L_{\text{unsup}}, \end{array}$$ (1)

where $L_{\text{sup}}$ measures the segmentation error against available annotations, $L_{\text{unsup}}$ enforces prediction consistency or feature regularization on unlabeled data, and λ controls the balance between the two losses.

In summary, the task aims to leverage the complementary strengths of limited labeled and abundant unlabeled 3D medical images to achieve accurate and robust segmentation performance. This semi-supervised strategy is particularly valuable for clinical applications, where annotated datasets are scarce, but large volumes of unlabeled MRI or PET images are readily available.

### 2.2 Overview of SemiSAM

Semi-supervised 3D medical image segmentation (SSMIS) aims to effectively exploit both limited labeled data and abundant unlabeled data to enhance segmentation performance. A representative paradigm in this domain is the Mean Teacher framework (40), which maintains two networks with identical architectures but different update strategies: a *student model* and a *teacher model*. The student model is directly optimized by gradient descent on the labeled data using a supervised loss $L_{\text{sup}}$, while the teacher model serves as a temporal ensemble of the student model, updated by an exponential moving average (EMA) of its weights (41). For each unlabeled image, the teacher model generates pseudo-labels ỹ, which are then used to enforce prediction consistency between the student and teacher networks. This strategy encourages smooth and stable decision boundaries in the feature space, effectively leveraging unlabeled data to improve model generalization. However, when labeled data are extremely scarce, the pseudo-labels generated by the teacher model can be inaccurate or incomplete, limiting performance gains (42). To address this issue, SemiSAM (34) introduces a foundation model-driven enhancement by integrating the Segment Anything Model (SAM) into the Mean Teacher framework. Specifically, the teacher model produces coarse segmentation maps on unlabeled data, which are used to generate point- or box-based prompts for SAM. Leveraging its strong zero-shot generalization capability, SAM produces refined pseudo-labels ỹ_SAM_ that provide more reliable supervision signals. These SAM-derived pseudo-labels are incorporated into the training pipeline through an additional regularization term that aligns the student model's prediction with SAM's output:


$$\begin{array}{l} L_{\text{total}}=L_{\text{sup}}+\lambda_{1}L_{\text{con}}+\lambda_{2}L_{\text{SAM}}, \end{array}$$ (2)

where $L_{\text{SAM}}$ enforces consistency between the segmentation network and SAM predictions, and λ_1_, λ_2_ are weighting coefficients.

By combining the complementary strengths of teacher-student consistency learning and SAM's generalizable segmentation priors, SemiSAM enhances pseudo-label quality and stabilizes training under low-annotation regimes. This makes the framework particularly effective in challenging clinical scenarios, such as when only one or a few labeled 3D volumes are available, significantly improving both the accuracy and robustness of semi-supervised medical image segmentation.

### 2.3 Proposed multi-view collaborative framework

**Figure 1:**
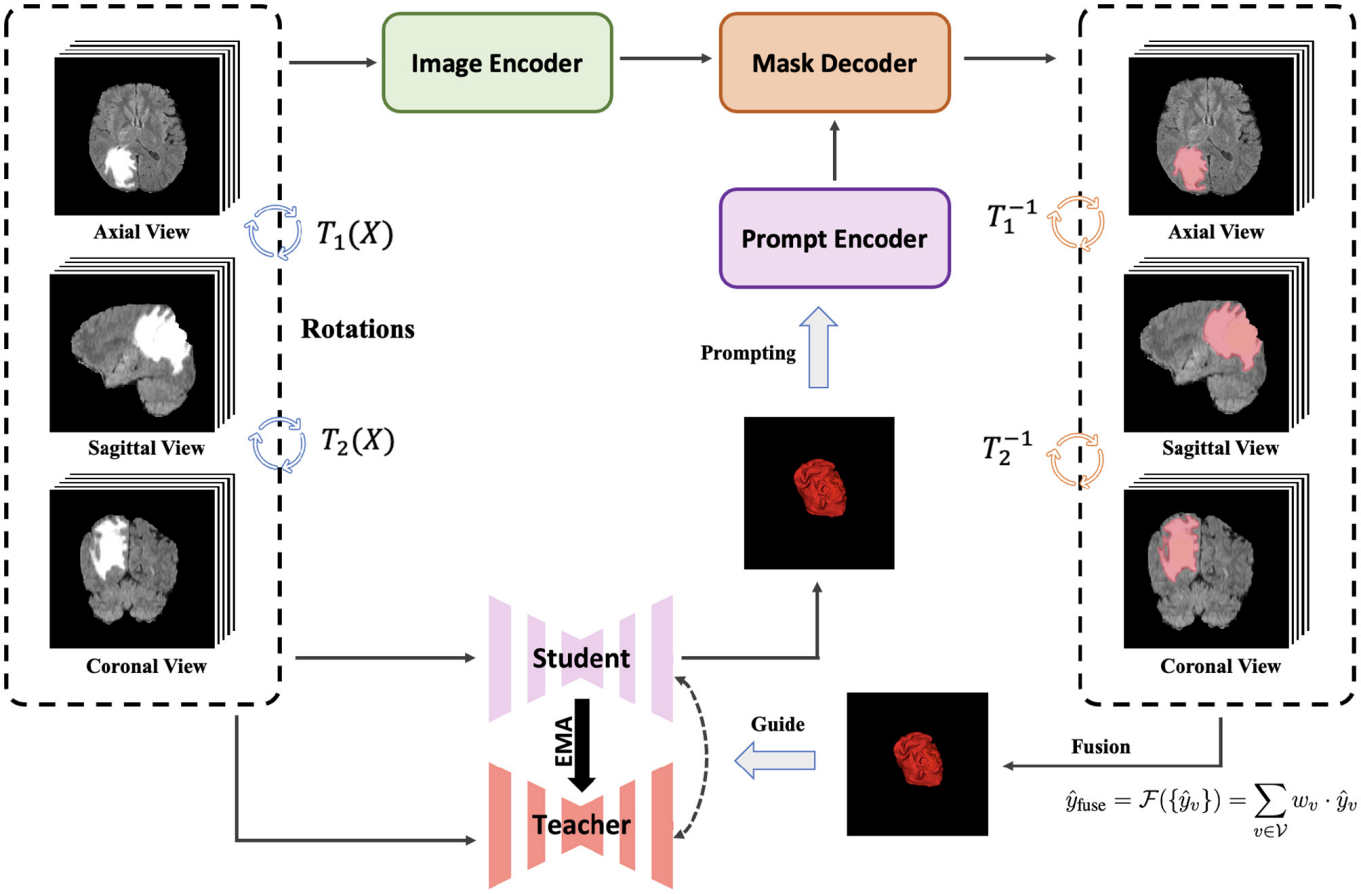
The overall architecture proposed multi-view collaborative SemiSAM (MVC-SemiSAM) framework. The framework utilizes SAM-like foundation model for axial, sagittal and coronal planes for fusion of different features to enhances the learning process. We aim to enhance the quality of pseudo labels to improve the overall performance in the learning procedure.

In 3D medical imaging, anatomical structures can be visualized from multiple orthogonal planes, typically the axial, sagittal, and coronal views. Each view provides complementary contextual and structural information that contributes to a more complete understanding of the underlying anatomy. To effectively exploit this multi-view information, we propose a multi-view collaborative SemiSAM (MVC-SemiSAM) framework, as illustrated in Figure 1.

Building upon the SemiSAM paradigm, MVC-SemiSAM extends the segmentation process from a single-view setting to a collaborative multi-view learning scheme. Specifically, separate SemiSAM branches are established for each view, denoted as *v*∈{axial, sagittal, coronal}. Each branch performs independent inference with view-specific prompts and pseudo-label generation, enabling the model to capture unique textural and spatial cues present in that particular orientation. This design preserves the complementary nature of different projections while maintaining the independence necessary for robust feature learning.

After obtaining view-specific segmentation outputs ŷ_*v*_, MVC-SemiSAM introduces a collaborative fusion mechanism to aggregate these predictions and generate unified supervision for the mean teacher framework. The multi-view fusion is expressed as:


$${\hat{y}}_{\text{fuse}}=ℱ(\{{\hat{y}}_{v}\})=\sum_{v\in V}w_{v}\cdot {\hat{y}}_{v},\text{ where }V=\{\text{axial},\text{sagittal},\text{coronal}\}$$ (3)

where F(·) denotes a view-collaboration function that integrates information across all views via averaging, confidence-weighted fusion, or attention-based refinement. This fused prediction is then used to guide both the student and teacher models through consistency constraints, encouraging the network to maintain cross-view coherence and spatial continuity. Accordingly, the total loss function for MVC-SemiSAM can be formulated as


$$L_{\text{total}}=L_{\text{sup}}(f_{\theta }(x),y)+\lambda_{1}L_{\text{con}}(f_{\theta }(x),f_{\theta '}(x))+\lambda_{2}L_{\text{SAM}}({\hat{y}}_{\text{fuse}},f_{\theta }(x)),$$ (4)

where $L_{\text{sup}}$ is the supervised loss on labeled data, $L_{\text{con}}$ is the consistency loss with the teacher model, $L_{\text{SAM}}$ enforces agreement between the student's prediction and the multi-view fused pseudo-label ŷ_fuse_, and λ_1_, λ_2_ are weighting coefficients.

By leveraging complementary information across orthogonal planes, MVC-SemiSAM effectively mitigates the influence of artifacts, noise, or partial occlusions that may occur in a single view. The collaborative learning strategy also enhances pseudo-label reliability and promotes volumetric consistency during semi-supervised training. In this way, the proposed framework achieves more accurate and stable segmentation results across 3D medical imaging modalities.

## 3 Results

### 3.1 Dataset and evaluation metrics

**Figure 2:**
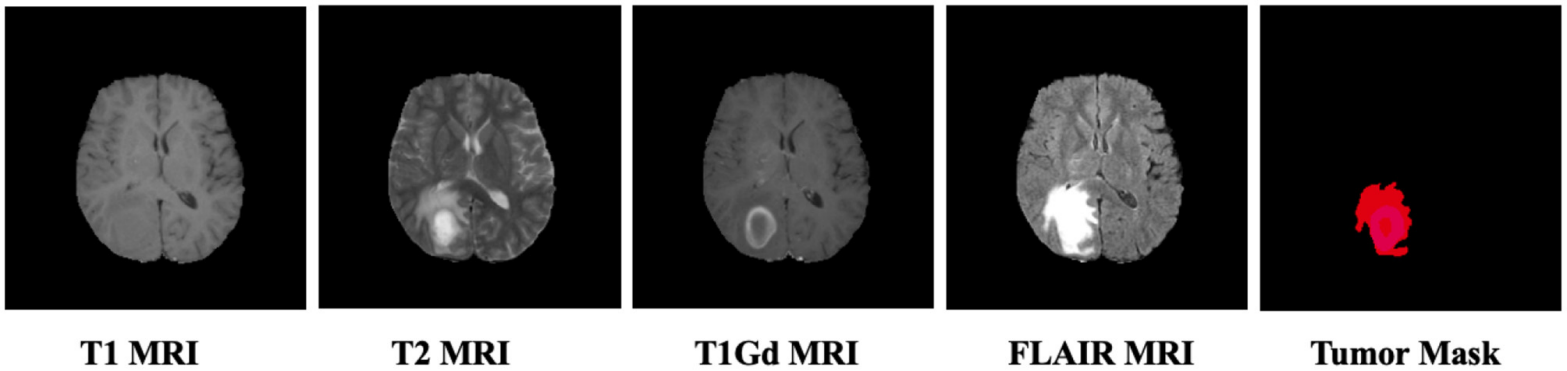
Comparison of different MRI modalities with corresponding annotation mask for brain tumor segmentation.

Our proposed method is evaluated on two different datasets. The first dataset is the Brain Tumor Segmentation (BraTS) 2019 dataset (39). The dataset contains multi-institutional preoperative MRI of 335 glioma patients, where each patient has four modalities of MRI scans including T1, T1Gd, T2, and FLAIR with neuroradiologist-examined labels. An example of the dataset is shown in Figure 2. For our research, we focus on the semi-supervised segmentation of whole tumors using FLAIR MRI images, as FLAIR images are particularly effective in characterizing malignant tumors due to their ability to highlight areas of brain edema and tumor infiltration, making them a preferred modality for this task (43). The second dataset is the PET heart segmentation derived from AutoPET-Organ (9), which contains 100 FDG-PET images with expert-examined annotations of the heart for quantitative evaluation of the cardiac metabolism. All the scans are resampled to the same resolution of 1 × 1 × 1 mm^3^ with intensity normalized to zero mean and unit variance. In our experiments, we split the MRI dataset into 250 scans for training, 25 scans for validation and the remaining 60 scans for testing, and the PET dataset into 40 scans for training, 10 scans for validation, and 50 scans for testing. Among the training scans, we follow the design of Zhang et al. (42) using the same 1/2/3/5 scans as labeled data and the remaining scans as unlabeled data. To effectively balance the supervised segmentation loss and the consistency regularization terms, we employ time-dependent weighting strategies utilizing a sigmoid-like ramp-up weighting coefficient to mitigate the disturbance of consistency loss during the early training stages as $\lambda_{1}=0.1\cdot e^{-5\left(1-t/t_{\text{max}}\right)}$, and a ramp-down weighting coefficient to leverage the strong zero-shot capabilities of the foundation model while preventing potential negative transfer in later stages as $\lambda_{2}=0.1\cdot e^{-5\left(t/t_{\text{max}}\right)}$, where *t* represents the current iteration number, and *t*_max_ represents the maximum number of iterations. We utilize average fusion of results from multiple view, where *w*_*v*_ = 1/3. To quantitatively evaluate the segmentation results, we use four complementary evaluation metrics. Dice similarity coefficient (Dice) and Jaccard Index (Jaccard), two region-based metrics, are used to measure the region mismatch. Average surface distance (ASD) and 95% Hausdorff Distance (95HD), two boundary-based metrics, are used to evaluate the boundary errors between the segmentation results and the ground truth.

### 3.2 Implementation details

To implement the SSMIS for brain tumor segmentation, we use the official codebase of SemiSAM+ in our experiments. Following the same setting, we use SAM-Med3D (44) as the SAM backbone. We use the Stochastic Gradient Descent (SGD) optimizer to update the network parameters with an initial learning rate of 0.01 decayed by 0.1 every 2,500 iterations. The maximum training iterations is set to 6,000. The batch size is set to 2, consisting of one labeled images and one unlabeled images in each mini-batch. We randomly crop 128 × 128 × 128 sub-volumes as the network input and the final segmentation results are obtained using a sliding window strategy. We use the standard data augmentation techniques on-the-fly to avoid overfitting during the training procedure, including randomly flipping, and rotating with 90, 180, and 270 degrees along the axial plane (38).

### 3.3 Experimental setup

To validate the effectiveness of our proposed framework, we compare it against different SSMIS implementations. All methods were evaluated under the same protocol using 1, 2, 3, and 5 labeled scans, respectively, with the remainder of the training scans serving as unlabeled data. The compared methods include (1) baseline: a standard supervised model trained using only the limited labeled data, without any SSL or SAM-based components. This represents the lower bound performance. (2) w/o SemiSAM: this is the classic Mean Teacher framework (40), a widely-used SSL method. It serves to quantify the performance gain from a standard SSL approach that leverages unlabeled data through consistency regularization. (3) SemiSAM (S/A/C): this represents the SemiSAM method (34), where the Mean Teacher framework is augmented with pseudo-labels generated by the Segment Anything Model (SAM). To establish strong baselines and for the purpose of ablation study, we apply SemiSAM independently on each of the three anatomical 3D views: Sagittal (S), Axial (A), and Coronal (C). (4) MVC-SemiSAM: our proposed Multi-view Collaborative SemiSAM framework. This method ensembles the outputs from the three independent view-specific SemiSAM branches to collaboratively guide the learning of the mean teacher framework, aiming to produce more robust and accurate segmentations.

### 3.4 Experimental results on MRI dataset

**Figure 3:**
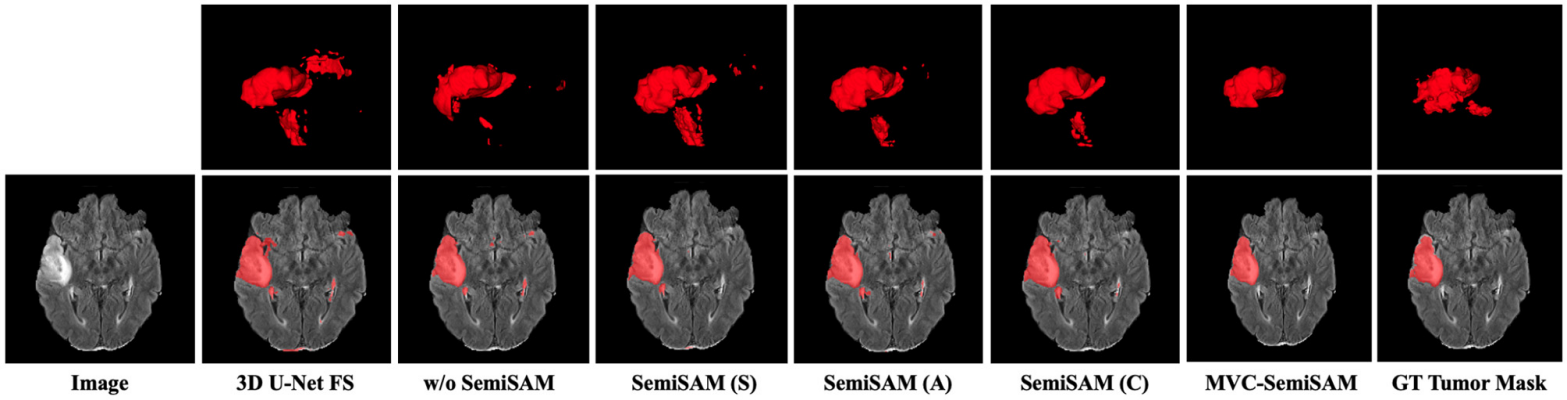
Visual comparison of segmentation results of different methods for MRI brain tumor segmentation.

Table 1 summarizes the quantitative performance of all compared methods under varying numbers of labeled cases on MRI brain tumor segmentation task. Overall, MVC-SemiSAM consistently outperforms baseline and existing SemiSAM variants across all metrics, demonstrating the effectiveness of multi-view collaborative learning in semi-supervised 3D segmentation. With only one labeled case, the baseline model achieves a Dice score of 42.82% and a Jaccard index of 29.01%, indicating poor segmentation performance due to extremely limited supervision. Incorporating SemiSAM significantly improves performance, with Dice scores reaching 67%–68% and Jaccard indices around 53%–56%, reflecting the benefit of SAM-generated pseudo-labels in providing additional supervision. Among these, MVC-SemiSAM further improves the Dice score to 71.63% and the Jaccard index to 59.50%, while simultaneously reducing the 95HD to 33.63 voxels and ASD to 13.52 voxels. These results suggest that multi-view fusion not only enhances overlap-based accuracy but also improves boundary delineation, mitigating the effects of noise or ambiguous structures present in a single view. As the number of labeled cases increases, all methods exhibit gradual performance improvements. With two labeled cases, MVC-SemiSAM achieves a Dice of 74.35% and a Jaccard of 62.29%, outperforming the best SemiSAM variant by approximately 1%–2%. Notably, the boundary-based metrics (95HD 23.45 voxels, ASD 8.07 voxels) also indicate more precise contour delineation, highlighting the model's ability to maintain volumetric consistency across the three views. This trend continues with three and five labeled cases, where MVC-SemiSAM achieves Dice scores of 76.08 and 77.91%, respectively, while keeping 95HD and ASD consistently lower than all baselines. Comparing single-view SemiSAM variants (S, A, C) to MVC-SemiSAM, it is evident that multi-view collaboration contributes substantial gains. While the single-view variants already improve pseudo-label quality via SAM, they are still susceptible to view-specific artifacts or local ambiguities. The multi-view fusion in MVC-SemiSAM effectively aggregates complementary information from axial, sagittal, and coronal views, resulting in more reliable and consistent segmentation predictions. Figure 3 presents the visual comparison of brain tumor segmentation results obtained by different methods on representative MRI slices. From left to right are the input image, results of 3D U-Net (fully supervised baseline), Mean Teacher without SAM (w/o SemiSAM), single-view SemiSAM from sagittal (S), axial (A), and coronal (C) planes, our proposed MVC-SemiSAM, and the ground truth (GT) tumor mask. The highlighted red regions indicate the predicted tumor areas. It can be observed that MVC-SemiSAM produces more accurate and complete tumor boundaries, closely matching the ground truth, while reducing false positives and missing regions compared to other models.

**Table 1:** The quantitative performance of all compared methods on MRI brain tumor segmentation.

**Evaluation metrics**					
**Method**	**Labeled**	**Dice (%)** ↑	**Jaccard (%)** ↑	**95HD (voxel)** ↓	**ASD (voxel)** ↓
Baseline	1 case	42.82 (18.13)	29.01 (15.43)	63.55 (17.71)	30.43 (11.88)
w/o SemiSAM	1 case	52.56 (15.56)	37.13 (14.51)	58.80 (21.11)	25.41 (13.78)
MVC w/o SemiSAM	1 case	69.01 (22.29)	56.79 (24.37)	36.58 (30.48)	15.45 (16.22)
SemiSAM (S)	1 case	68.53 (22.22)	56.22 (24.39)	38.81 (29.05)	16.66 (15.93)
SemiSAM (A)	1 case	67.46 (17.24)	53.33 (18.87)	41.42 (29.32)	15.81 (15.58)
SemiSAM (C)	1 case	67.68 (17.05)	53.56 (18.79)	41.15 (29.32)	15.76 (15.53)
MVC-SemiSAM	1 case	71.63 (20.73)	59.50 (23.14)	33.63 (29.26)	13.52 (15.15)
Baseline	2 cases	53.70 (18.77)	38.98 (17.89)	77.37 (25.85)	33.57 (15.33)
w/o SemiSAM	2 cases	66.16 (18.64)	52.21 (20.13)	45.73 (29.11)	19.07 (16.02)
MVC w/o SemiSAM	2 cases	69.88 (20.86)	57.40 (23.22)	34.77 (30.29)	14.70 (15.92)
SemiSAM (S)	2 cases	72.59 (18.57)	60.04 (21.22)	37.57 (28.71)	14.32 (15.04)
SemiSAM (A)	2 cases	73.27 (16.20)	60.23 (19.04)	25.73 (27.43)	8.91 (13.08)
SemiSAM (C)	2 cases	73.83 (16.47)	61.02 (19.27)	25.33 (27.41)	9.23 (13.46)
MVC-SemiSAM	2 cases	74.35 (16.43)	62.29 (19.15)	23.45 (26.23)	8.07 (12.95)
Baseline	3 cases	56.06 (24.68)	42.81 (22.83)	47.67 (20.56)	17.52 (12.94)
w/o SemiSAM	3 cases	68.11 (18.60)	54.45 (20.10)	36.57 (29.65)	12.85 (15.45)
MVC w/o SemiSAM	3 cases	73.34 (21.99)	61.87 (23.14)	22.33 (25.81)	8.18 (13.85)
SemiSAM (S)	3 cases	73.92 (21.26)	62.51 (23.28)	19.77 (21.89)	6.13 (9.76)
SemiSAM (A)	3 cases	73.77 (19.14)	61.72 (21.84)	23.48 (26.34)	7.58 (13.32)
SemiSAM (C)	3 cases	74.14 (18.42)	61.98 (21.23)	24.36 (28.04)	8.01 (13.59)
MVC-SemiSAM	3 cases	76.08 (17.99)	64.40 (20.85)	20.14 (24.65)	6.71 (13.03)
Baseline	5 cases	62.35 (26.60)	50.16 (25.54)	36.68 (31.61)	11.83 (17.33)
w/o SemiSAM	5 cases	70.48 (20.83)	57.91 (21.97)	35.79 (29.14)	11.83 (11.30)
MVC w/o SemiSAM	5 cases	73.70 (20.63)	62.00 (22.55)	21.06 (25.05)	7.73 (12.37)
SemiSAM (S)	5 cases	76.00 (15.86)	63.72 (19.18)	25.82 (26.15)	8.89 (12.64)
SemiSAM (A)	5 cases	76.89 (18.00)	65.47 (20.81)	20.49 (24.28)	7.45 (13.16)
SemiSAM (C)	5 cases	76.80 (18.19)	65.40 (20.92)	19.69 (24.20)	7.30 (12.95)
MVC-SemiSAM	5 cases	77.91 (17.10)	66.23 (19.93)	18.49 (22.76)	6.47 (13.52)

### 3.5 Experimental results on PET dataset

Following the MRI-based experiments, we further evaluate the generalization capability of our proposed MVC-SemiSAM framework on the whole-body PET heart segmentation task. The quantitative results summarized in Table 2 reveal several consistent and noteworthy findings. First, similar to the MRI results, both semi-supervised learning (SSL) and SAM-based supervision demonstrate clear advantages over the fully supervised baseline trained with limited labeled data. The incorporation of unlabeled PET scans enables the Mean Teacher framework (w/o SemiSAM) to achieve a substantial improvement in Dice and Jaccard scores, confirming that semi-supervised consistency regularization effectively alleviates overfitting in low-annotation regimes. Second, introducing SAM-generated pseudo-labels through the SemiSAM variants further enhances segmentation performance, particularly in terms of region completeness and boundary accuracy. The improvement is especially pronounced in the extremely low-label settings (e.g., one or two labeled cases), where foundation model guidance helps the network capture organ boundaries that are difficult to infer from limited labeled examples alone. Most importantly, our proposed MVC-SemiSAM achieves the highest performance across nearly all metrics and annotation levels. The collaborative fusion of multi-view predictions yields more stable and coherent segmentation results, as evidenced by consistently higher Dice and Jaccard scores and reduced boundary errors (95HD and ASD). This validates the robustness of the proposed framework in handling modality-specific noise and anatomical variability inherent in PET imaging. Overall, the PET experiments further confirm the universality and transferability of MVC-SemiSAM across different 3D medical imaging modalities. By effectively leveraging unlabeled data and multi-view structural information, the framework provides a scalable solution applicable to both anatomical (MRI) and functional (PET) segmentation tasks.

**Table 2:** The quantitative performance of all compared methods on PET heart segmentation.

**Evaluation metrics**					
**Method**	**Labeled**	**Dice (%)** ↑	**Jaccard (%)** ↑	**95HD (voxel)** ↓	**ASD (voxel)** ↓
Baseline	1 case	26.96 (11.88)	15.98 (8.19)	119.74 (20.49)	34.48 (8.15)
w/o SemiSAM	1 case	32.90 (16.95)	20.95 (12.44)	49.64 (41.76)	18.40 (14.93)
MVC w/o SemiSAM	1 case	40.31 (17.13)	26.72 (13.88)	55.40 (28.85)	22.43 (11.22)
SemiSAM (S)	1 case	38.69 (21.35)	25.82 (16.37)	33.85 (43.36)	17.20 (15.25)
SemiSAM (A)	1 case	38.83 (22.77)	26.64 (18.22)	21.41 (26.82)	7.56 (11.18)
SemiSAM (C)	1 case	45.20 (18.74)	31.13 (16.10)	30.75 (30.88)	11.53 (8.86)
MVC-SemiSAM	1 case	48.47 (20.31)	34.29 (17.34)	45.38 (43.78)	15.82 (13.39)
Baseline	2 cases	28.97 (10.82)	17.72 (7.91)	108.67 (32.59)	32.29 (12.87)
w/o SemiSAM	2 cases	37.88 (15.71)	24.57 (12.60)	52.71 (40.52)	16.87 (9.68)
MVC w/o SemiSAM	2 cases	50.44 (16.85)	35.32 (14.24)	71.09 (49.76)	20.14 (13.58)
SemiSAM (S)	2 cases	54.48 (20.93)	39.95 (17.61)	79.42 (31.50)	24.28 (12.90)
SemiSAM (A)	2 cases	61.80 (11.73)	45.68 (11.44)	67.51 (40.39)	17.46 (10.74)
SemiSAM (C)	2 cases	57.07 (12.64)	41.00 (12.15)	49.04 (51.82)	15.21 (14.12)
MVC-SemiSAM	2 cases	64.85 (14.87)	49.56 (14.35)	12.05 (20.48)	4.40 (4.08)
Baseline	3 cases	31.07 (9.87)	18.74 (6.70)	100.06 (31.44)	31.84 (22.61)
w/o SemiSAM	3 cases	49.77 (12.30)	33.99 (10.56)	78.84 (39.08)	21.36 (10.35)
MVC w/o SemiSAM	3 cases	53.48 (14.85)	37.84 (13.26)	94.86 (42.21)	31.46 (16.43)
SemiSAM (S)	3 cases	60.37 (14.47)	44.64 (13.60)	63.56 (42.70)	17.03 (9.82)
SemiSAM (A)	3 cases	65.75 (13.41)	50.30 (13.28)	50.85 (46.44)	13.91 (9.24)
SemiSAM (C)	3 cases	65.23 (8.71)	48.99 (9.28)	70.32 (35.23)	19.59 (10.01)
MVC-SemiSAM	3 cases	67.46 (9.38)	51.62 (10.18)	72.20 (33.17)	19.00 (10.21)
Baseline	5 cases	34.56 (12.73)	21.98 (10.30)	97.57 (28.29)	30.31 (12.52)
w/o SemiSAM	5 cases	63.02 (11.24)	46.93 (11.25)	40.57 (51.61)	12.95 (12.25)
MVC w/o SemiSAM	5 cases	66.70 (16.56)	49.45 (14.34)	43.06 (45.27)	13.35 (14.17)
SemiSAM (S)	5 cases	67.10 (10.49)	51.32 (10.56)	11.13 (20.57)	3.46 (4.00)
SemiSAM (A)	5 cases	68.48 (8.75)	52.71 (9.65)	23.56 (47.31)	7.50 (12.68)
SemiSAM (C)	5 cases	66.13 (15.58)	51.10 (14.71)	13.25 (25.30)	3.77 (4.31)
MVC-SemiSAM	5 cases	71.67 (10.62)	56.76 (11.10)	19.36 (37.51)	6.94 (11.68)

**Figure 4:**
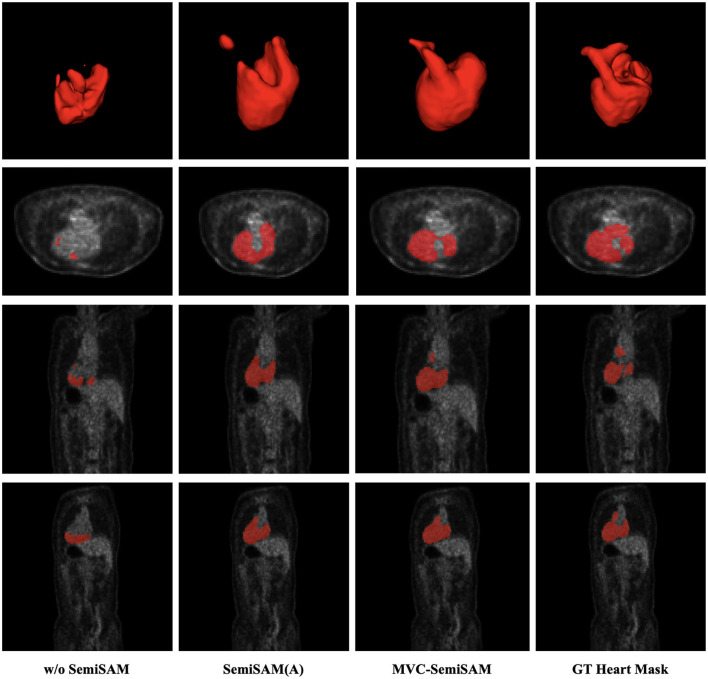
Visual comparison of segmentation results of different methods for PET heart segmentation.

Figure 4 illustrates the qualitative comparison of different methods on representative PET heart segmentation examples. Consistent with the quantitative results, the proposed MVC-SemiSAM produces segmentation maps that are visually closest to the ground truth, achieving more complete and coherent heart boundaries. These qualitative improvements highlight the effectiveness of the proposed multi-view collaborative mechanism in refining SAM-guided pseudo-labels and leveraging cross-view consistency to achieve anatomically precise PET segmentation. Together with the MRI findings, these results demonstrate the robustness and modality adaptability of MVC-SemiSAM for both structural and functional medical imaging tasks.

## 4 Discussion

This study investigates the integration of foundation model priors and semi-supervised learning for efficient 3D medical image segmentation under limited annotation scenarios. The experimental findings reveal several key insights into the advantages and generalizability of the proposed framework.

First, consistency-based SSL proves to be a reliable strategy for mitigating the dependence on extensive manual annotations (40). By jointly optimizing predictions from labeled and unlabeled data, the framework enhances the robustness and generalization of segmentation models, which is particularly valuable for clinical applications where annotation resources are scarce. The observed improvement over purely supervised baselines demonstrates the effectiveness of SSL in harnessing abundant unlabeled medical scans to improve 3D segmentation quality (15). Integrating foundation models like the Segment Anything Model (SAM) (31) into the semi-supervised learning pipeline introduces an additional layer of generalist prior knowledge. As foundation models are trained on large-scale annotated data, they provides shape priors, boundary cues, and prompt-driven flexibility that complement the limited domain-specific supervision available in medical datasets (44). Through pseudo-label generation and consistency constraints, SAM acts as an auxiliary teacher, effectively transferring its structural awareness into the medical domain. This cross-domain supervision leads to more anatomically coherent predictions, especially in regions with ambiguous intensity contrast or irregular boundaries (32).

The core innovation of our framework lies in its multi-view collaborative learning mechanism, which fully leverages the inherent 3D nature of volumetric imaging. By processing sagittal, coronal, and axial planes independently and fusing their predictions, the framework captures complementary spatial information and mitigates artifacts or ambiguities that may arise from a single projection. This collaborative fusion substantially enhances model stability and improves boundary precision, demonstrating the value of multi-view reasoning for complex anatomical structures. Importantly, the benefits of MVC-SemiSAM extend beyond a specific organ or imaging modality. The consistent performance across both anatomical MRI and functional PET modalities highlights its modality-agnostic and task-general nature. This suggests that MVC-SemiSAM is not limited to neuroimaging but can serve as a unified strategy for 3D medical image segmentation. Moreover, the frameworks modular design allows seamless adaptation to other semi-supervised paradigms and foundation models. Future iterations could integrate domain-specific vision-language models or multi-modal priors to further enhance generalization and interpretability. By bridging the gap between foundation model priors and domain-aware multi-view reasoning, MVC-SemiSAM offers a promising direction toward universal, label-efficient segmentation solutions in medical imaging.

## 5 Conclusion

This study introduces a foundation model-driven, multi-view collaborative learning framework that advances semi-supervised 3D medical image segmentation under extremely limited annotation conditions. By synergistically combining the generalist prior knowledge of SAM, the data efficiency of consistency-based semi-supervised learning, and the inherent robustness of multi-view representation, MVC-SemiSAM provides a label-efficient yet highly accurate segmentation framework. Validated on both MRI and PET modalities, the proposed method demonstrates strong adaptability and clinical relevance, reducing] annotation dependency while maintaining reliable segmentation quality across different imaging domains. Its modular and scalable design offers a practical foundation for future extensions, such as integrating multimodal data or domain-adaptive foundation models for universal medical image understanding.

## Data Availability

Publicly available datasets were analyzed in this study. This data can be found at: https://www.med.upenn.edu/cbica/brats-2019/.
